# Histamine deficiency facilitates coronary microthrombosis after myocardial infarction by increasing neutrophil‐platelet interactions

**DOI:** 10.1111/jcmm.15037

**Published:** 2020-02-16

**Authors:** Hui Li, Chao Tang, Xiaowei Zhu, Weiwei Zhang, Mieradilijiang Abudupataer, Suling Ding, Caiwen Duan, Xiangdong Yang, Junbo Ge

**Affiliations:** ^1^ Shanghai Institute of Cardiovascular Diseases Zhongshan Hospital Fudan University Shanghai China; ^2^ Department of Cardiology Zhongshan Hospital Fudan University Shanghai China; ^3^ Key Laboratory of Pediatric Hematology and Oncology Ministry of Health Shanghai JiaoTong University School of medicine (SJTU‐SM) Shanghai China; ^4^ Pediatric Translational Medicine Institute Shanghai Children's Medical Center Shanghai Collaborative Innovation Center for Translational Medicine Shanghai JiaoTong University School of medicine (SJTU‐SM) Shanghai China; ^5^ Department of Pharmacology and Chemical Biology Shanghai JiaoTong University School of medicine (SJTU‐SM) Shanghai China; ^6^ Department of Cardiac Surgery Zhongshan Hospital Fudan University Shanghai China; ^7^ Institutes of Biomedical Sciences Fudan University Shanghai China

**Keywords:** acute myocardial infarction, coronary microthrombosis, histamine, neutrophil‐platelet interactions

## Abstract

Neutrophil‐platelet interactions are responsible for thrombosis as well as inflammatory responses following acute myocardial infarction (AMI). While histamine has been shown to play a crucial role in many physiological and pathological processes, its effects on neutrophil‐platelet interactions in thromboinflammatory complications of AMI remain elusive. In this study, we show a previously unknown mechanism by which neutrophil‐derived histamine protects the infarcted heart from excessive neutrophil‐platelet interactions and redundant arterial thrombosis. Using histamine‐deficient (histidine decarboxylase knockout, HDC^−/−^) and wild‐type murine AMI models, we demonstrate that histamine deficiency increases the number of microthrombosis after AMI, in accordance with depressed cardiac function. Histamine‐producing myeloid cells, mainly Ly6G^+^ neutrophils, directly participate in arteriole thrombosis. Histamine deficiency elevates platelet activation and aggregation by enhancing Akt phosphorylation and leads to dysfunctional characteristics in neutrophils which was confirmed by high levels of reactive oxygen species production and CD11b expression. Furthermore, HDC^−/−^ platelets were shown to elicit neutrophil extracellular nucleosomes release, provoke neutrophil‐platelet interactions and promote HDC‐expressing neutrophils recruitment in arteriole thrombosis in vivo. In conclusion, we provide evidence that histamine deficiency promotes coronary microthrombosis and deteriorates cardiac function post‐AMI, which is associated with the enhanced platelets/neutrophils function and neutrophil‐platelet interactions.

## 1 INTRODUCTION

Neutrophil‐platelet interactions play a crucial role in thromboinflammatory complications of cardiovascular diseases.^1^, ^2^ Myocardial injury triggers acute inflammatory responses in the ischaemic region, which not only promotes clearance of debris and tissue repair, but leads to deterioration of cardiac function and heart failure.^3^ Coronary microthrombosis in infarct‐related arteries largely affects functional recovery of viable muscle, infarct expansion and healing process of acute myocardial infarction (AMI).^4^ Histological analysis has shown that neutrophils are enriched in arterial thrombi of AMI patients.^5^, ^6^ Activated P‐selectin^+^ platelets bind to circulating neutrophils, which leads to their recruitment to the injured endothelial surface, a process which is critical to the formation of neutrophil‐platelet aggregates and initiation of inflammatory responses.^7^, ^8^ These interactions also initiate the formation of neutrophil extracellular traps (NETs), web‐like networks of chromatin, histones and degradative enzymes, which in return induces platelet activation and initiates coagulation.^9^, ^10^, ^11^ Although the role of neutrophil‐platelet interactions in the regulation of thromboinflammation has been increasingly recognized, the mechanisms by which these interactions contribute to thrombosis and inflammation remain largely elusive.

Histidine decarboxylase (HDC), the key enzyme for histamine synthesis, is highly expressed in myeloid cells, particularly in CD11b^+^Gr‐1^+^ immature myeloid cells.^12^, ^13^ AMI elevates serum histamine level in patients with acute coronary syndrome, as well as in murine AMI model.^12^ Moreover, HDC‐enhanced green fluorescence protein (EGFP)^+^ myeloid cells abundantly infiltrate into the infarct area post‐AMI.^12^ Although neutrophils are widely believed to be an injury aggravator, recent studies have indicated that neutrophils possess anti‐inflammatory characteristics.^14^ Likewise, studies in histamine‐deficient (HDC knockout, HDC^−/−^) mice revealed that histamine derived from CD11b^+^ myeloid cells exerts a protective effect against cardiomyocyte apoptosis after AMI, rather than having a pro‐inflammatory function.^12^, ^15^ Furthermore, local tissue signals are thought to provide feedback to stem cells and therefore regulate the regeneration of immune cells.^16^ Intravital microscopy studies have revealed that tissue‐infiltrated neutrophils perform their repair functions and finally migrate back into the bone marrow.^17^ While the roles of neutrophils in thromboinflammatory responses have been gradually uncovered, the molecular signals linking neutrophil‐derived histamine with coronary thrombosis remain unknown.

In this study, we clarified that neutrophils are the main histamine‐releasing cells during coronary microthrombosis. Histamine deficiency results in functional alterations in both platelets and neutrophils and promotes neutrophil‐platelet interactions. These processes elicit neutrophil tethering and recruitment, increase the number of coronary microthrombosis and deteriorate cardiac function after AMI. Thus, our results provide insight into the pathogenic mechanisms underlying the protective effect of neutrophil‐derived histamine against the thromboinflammatory complications of AMI.

## 2 METHODS

Detailed Methods and Reagents are described in Appendix S1.

### 2.1 Mice and surgical procedures

Acute myocardial infarction surgery was carried out on mice as previously described.^12^ Briefly, mice were anaesthetized and intubated with a 22‐G intravenous catheter and then mechanically ventilated with 1.5%‐2.0% isoflurane gas using a rodent respirator. Left thoracotomy was performed at the fourth intercostal space, and the left anterior descending (LAD) coronary artery was ligated at 2‐3 mm from the tip of the left auricle using an 8‐0 silk suture. Ligation success was confirmed when the anterior wall of the left ventricle turned pale. Sham‐operated mice underwent identical procedures except that the suture placed under the LAD was not tied. The chest cavity was closed, and the animal was taken care of in a cage. In mice with reagent administration, histamine (4 mg/kg), pyrilamine (10 mg/kg) and cimetidine (10 mg/kg) were administered intraperitoneally (i.p.) daily beginning from 3 days before surgery and continuing until euthanasia. All controls were age‐ and sex‐matched. HDC^−/−^ and HDC‐EGFP mice were generously provided by Professor Timothy C. Wang from Columbia University. The absence of histamine was corroborated with immunofluorescence assays (Figure S1A). Wild‐type (WT) mice were obtained from the Department of Laboratory Animal Science, Fudan University. All animal procedures were in accordance with the guidelines from Directive 2010/63/EU of the European Parliament and were approved by the Committee on the Ethics of Animal Experiments of Fudan University. Mice were anaesthetized via inhalation of isoflurane gas (1.5%‐2.0%) or injection of pentobarbital (60 mg/kg, i.p.) for surgery and in vivo experiments, and killed via overdose of pentobarbital (150 mg/kg, i.p.). Reagent information is detailed in Table S1.

### 2.2 Transthoracic echocardiography

Transthoracic echocardiography (Vevo770 ultrasound systems; VisualSonics) was performed under anaesthesia. The left ventricular dimensions were quantified via digitally recorded two‐dimensional short‐axis M‐mode tracings at the level of the papillary muscles, allowing for consistent measurement at the same anatomic location in different mice. The left ventricular ejection fraction (LVEF) was calculated, and at least five consecutive beats were evaluated.

### 2.3 Histological analysis

Hearts were gently perfused with saline, fixed in 4% formaldehyde, embedded in paraffin, cut with a Leica RM2135 histotome into 5 μm sections and stained with haematoxylin‐eosin (H&E) to detect coronary microthrombi. Histological images were obtained using a Leica DM2500 light microscope and processed with the Leica Application Suite X (LASX) software. Quantitative analysis was carried out by manual counting of coronary arteriolar thrombi per section, with 3‐4 randomly chosen sections per sample.

### 2.4 Intravital microscopy of FeCl_3_‐induced thrombus formation in the mesenteric arteriole

DiD (cell‐labelling solution, Invitrogen, Cat No.V22887)‐labelled washed platelets collected from one donor mouse were injected into two HDC‐EGFP mice via the tail vein. Recipient mice were anaesthetized, the mesentery was exteriorized, and the chosen mesenteric arteriole was placed on a customized stage. A 1 × 3 mm strip of Whatman filter paper saturated with 10% FeCl_3_ solution was placed over the exposed mesenteric arteriole for 1 minute. Monitoring was initiated 1 minute before FeCl_3_ treatment and then continued for 27 minute (baseline 1 minute + treatment 1 minute + thrombus formation 25 minute). Analysis of leucocytes was carried out by evaluating the numbers of rolling and adherent cells in the visual field using a Leica M205 fluorescence microscope (magnification 130×). All scenes were recorded using a CCD camera (Leica DFC 7000T) and analysed with the LASX software. The mesenteric arterioles under observation ranged from 120‐200 μm in diameter as determined by the LASX software. Neutrophils with a recognizable displacement between two or more continuous images (time‐lapse interval 5.243 seconds) were determined as rolling neutrophils and those with an immobile position were considered adherent neutrophils.

### 2.5 FeCl_3_‐induced thrombus formation in carotid artery

Evaluation of FeCl_3_‐induced thrombus formation in carotid artery was performed as previously described.^18^ Briefly, mice were anaesthetized, and the carotid artery was carefully exposed. A laser Doppler system (Trimflo Fiber Optic Probe) was placed under the vessel to monitor the blood flow. A 1 × 3 mm strip of Whatman filter paper saturated with 10% FeCl_3_ solution was placed over the exposed carotid artery for 3 minute. The monitoring was initiated at the time of FeCl_3_ treatment and continued until occlusion. A carotid artery blood flow of <0.05 mL/min was considered occlusion. The carotid artery was then immediately excised, fixed in 4% paraformaldehyde, embedded in OCT medium (Sakura, Cat No.4583), cut with a Leica CM1850 histo‐cryotome into 10 μm sections and stored at −80°C.

### 2.6 Immunofluorescence analysis

Frozen samples were fixed with 4% paraformaldehyde, permeabilized with 0.1% Triton‐X‐100, blocked with 5% foetal bovine serum (Gibco, Cat No. 10099141C) and incubated with diluted (1:200) fluorochrome‐conjugated antibodies against mouse Ly‐6G (BD Biosciences, Cat No.551461), CD41 (Biolegend, Cat No.133913) or HDC (Abcam, Cat No. ab37291) overnight at 4°C. The nuclei were stained with DAPI‐containing mounting medium (Southern Biotech, Cat No.0100‐20). Images were acquired using a Leica SP8 confocal microscope and processed with the LASX software.

### 2.7 Blood cell count

Blood cell count was performed using EDTA‐anticoagulated blood with a veterinary haematology analyser (Mindray, BC‐5300Vet) according to the manufacturer's instructions.

### 2.8 Bleeding time

Mice were anaesthetized; then, a 5 mm segment of the tail was amputated, and the remaining tail was immediately immersed in saline at 37°C. The time required for the bleeding to spontaneously stop was measured.

### 2.9 Plasmatic coagulation analysis

Plasmatic coagulation parameters were assessed by thrombelastometry (ROTEM) according to the manufacturer's instructions. Prothrombin time (PT) was used as extrinsic activation test, and activated partial thromboplastin time (APTT) was used as intrinsic pathway analysis.

### 2.10 Flow cytometry analysis

Blood cell and bone marrow cells were harvested, erythrocytes were lysed with FACS lysing buffer (BD Biosciences, Cat No.349202), and the resuspended cells were labelled with diluted (1:200) antibodies. Antibody information is detailed in Table S2. Negative controls (fluorescence minus one, FMO) were used to determine positivity for all markers (Figure S4). Data were acquired using a FACS Canto flow cytometer (BD Biosciences) and processed with the FlowJo v10 software (Tree Star).

### 2.11 Wright‐Giemsa staining

Blood films were stained with the Wright‐Giemsa staining kit (Baso Biotech, Cat No.417033) according to the manufacturer's instructions.

### 2.12 Platelet functional assays

Platelet preparation, aggregation, spreading and clot retraction were processed as previously described^18^ and detailed in supplementary files.

### 2.13 Flow cytometry analysis of platelet activation

Washed platelets were stimulated with 0.05 U/mL thrombin for 20 minute at 37°C and labelled with fluorochrome‐conjugated antibodies against mouse P‐selectin, activated αIIbβ3 and CD41, and then fixed with 4% formaldehyde. In a separate set of experiments, platelets were incubated with histamine (10 μmol/L), pyrilamine (10 μmol/L) and cimetidine (10 μmol/L) for 20 minute before stimulation. Information regarding reagents and antibodies is detailed in Tables S1 and S2. Negative controls were used to define positivity for all markers (Figure S5A). Data were acquired using a FACS Canto flow cytometer (BD Biosciences) and processed with the FlowJo v10 software (Tree Star).

### 2.14 Neutrophil‐platelet interaction assays

Neutrophil preparation, neutrophil‐platelet aggregates formation and NETs formation in vitro were processed as previously described^19^ and detailed in supplementary files.

### 2.15 Statistical analysis

Data are expressed as mean ± standard error of mean (SEM). All data were normalized and analysed using the SPSS 23.0.0 statistics software (IBM). Student's *t* test was used for data evaluation between two groups, and one‐way analysis of variance (ANOVA, Bonferroni‐Dunn Correction) or two‐way ANOVA (Tukey method) was used for multiple comparisons. A *P* value of <.05 was considered statistically significant.

## 3 RESULTS

### 3.1 Histamine deficiency promotes microthrombosis and aggravates myocardial injury

**Figure 1:**
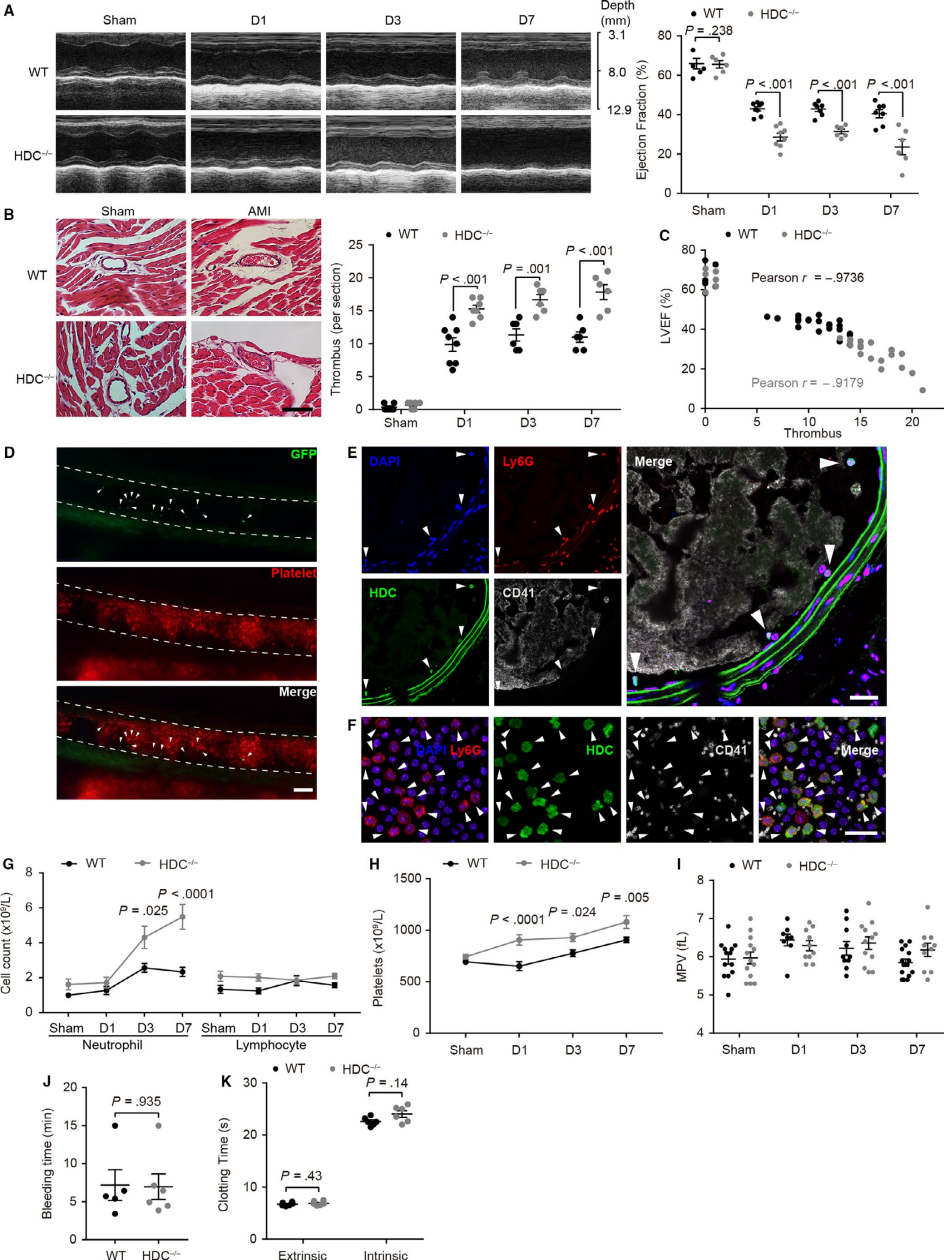
Histamine deficiency promotes microthrombosis and aggravates the myocardial injury. A, Representative images and quantitative analysis of cardiac function evaluated by left ventricular ejection fraction (LVEF). B, Representative images and quantitative analysis of haematoxylin‐eosin (H&E) stained coronary microthrombosis. Bar: 50 μm. C, Analysis of the correlation between LVEF and the numbers of microthrombi. D, Intravital microscopy images showing that platelets (red) and neutrophils (green, arrowheads) participate in FeCl_3_‐induced mesenteric arteriole thrombus. Bar: 100 μm. E, F, Immunofluorescence analysis. Representative images of (E) FeCl_3_‐induced carotid artery thrombus and (F) blood cells stained with Ly6G (neutrophils, red), GFP (HDC, green), CD41 (platelets, grey) and DAPI (nuclei, blue). Arrowheads show HDC‐positive neutrophils. Bar: 20 μm. Cell counts of (G) neutrophils, lymphocytes and (H) platelets in AMI mice. I, MPV, mean platelet volume. J, K, Haemostatic parameters. J, Tail bleeding time. K, Clotting time induced by either extrinsic or intrinsic activation of coagulation. Graphs show mean ± SEM. *P*‐values were determined by one‐way ANOVA with Bonferroni‐Dunn correction or Student's *t* test (J)

Permanent ligation of coronary LAD branch leads to severe myocardial injury and cardiac dysfunction. In accordance with previous reports,^12^ we found decreased cardiac function in HDC^−/−^ mice model compared to WT controls, which was confirmed by the LVEF value measured at day 1 (D1), day 3 (D3) and day 7 (D7) post‐surgery (Figure 1A). Blockage of histamine receptors (HRs) by pyrilamine (H1R‐selective inhibitor, H1Ri) or cimetidine (H2R‐selective inhibitor, H2Ri) in WT mice resulted in an LVEF reduction, especially in mice with blockage of histamine/H1R pathway, while histamine (HA) administration lessened the LVEF reduction in HDC^−/−^ mice (Figure S1B). Coronary microthrombosis is responsible for infarct expansion.^4^ We found that histamine deficiency increased the number of microthrombi in coronary arterioles (diameter ≤ 150 μm) of these infarcted hearts, which is consistent with the deteriorated cardiac function and increased infarct size^12^ (Figure 1B and Figure S1C). Moreover, the enhanced coronary microthrombosis induced by histamine deficiency is closely associated with impaired cardiac systole (Figure 1C).

Neutrophils are the first immune responders in acute inflammation.^13^ Previous studies have indicated that CD11b^+^Gr‐1^+^ myeloid cells residing in the bone marrow and spleen are the predominant HDC‐expressing cells, which may be activated and recruited to the inflamed tissue.^14^ Indeed, abundant HDC‐expressing myeloid cells infiltrate in the infarct area after AMI.^12^ To investigate whether HDC‐expressing myeloid cells participate in arterial thrombus formation, we generated chimeric mice by transplanting fluorochrome‐labelled platelets isolated from WT donor mice to HDC‐EGFP reporter mice (WT → HDC‐EGFP). FeCl_3_‐induced arterial injury triggers stable platelet‐rich thrombus formation in the lumen, thereby providing us with the opportunity to visualize the thrombosis process in vivo.^20^We identified the presence of HDC‐expressing myeloid cells in FeCl_3_‐induced mesenteric arteriole thrombosis and further confirmed that Ly6G^+^ neutrophils were predominant (Figure 1D‐F and Video S1). In addition, no EGFP signal was detected in platelets in either the artery thrombi, single platelets or megakaryocytes (Figure 1E,F and S1D), indicating no transcription or expression of *Hdc* gene in platelets.

In the peripheral blood, we found that neutrophils, rather than lymphocytes, are the primary reason for the increase in circulating leucocytes, while histamine deficiency led to a higher increase in circulating neutrophils after AMI (Figure 1G). Likewise, the myocardial injury resulted in an increased demand for platelets and histamine deficiency‐induced infarct‐related platelet enhancement (Figure 1H). Platelet size is strongly correlated with platelet function.^21^ We found that mean platelet volume (MPV) was neither affected by myocardial ischaemic stress or histamine deficiency (Figure 1I). Moreover, bleeding time and plasma clotting time were unchanged between HDC^−/−^ mice and WT mice (Figure 1J,K). Thus, these data suggest that histamine deficiency increases the number of circulating neutrophils and platelets after AMI, which may promote thromboinflammatory complications and myocardial injury.

### 3.2 Histamine ablation facilitates thrombocytopoiesis and granulopoiesis after AMI

**Figure 2:**
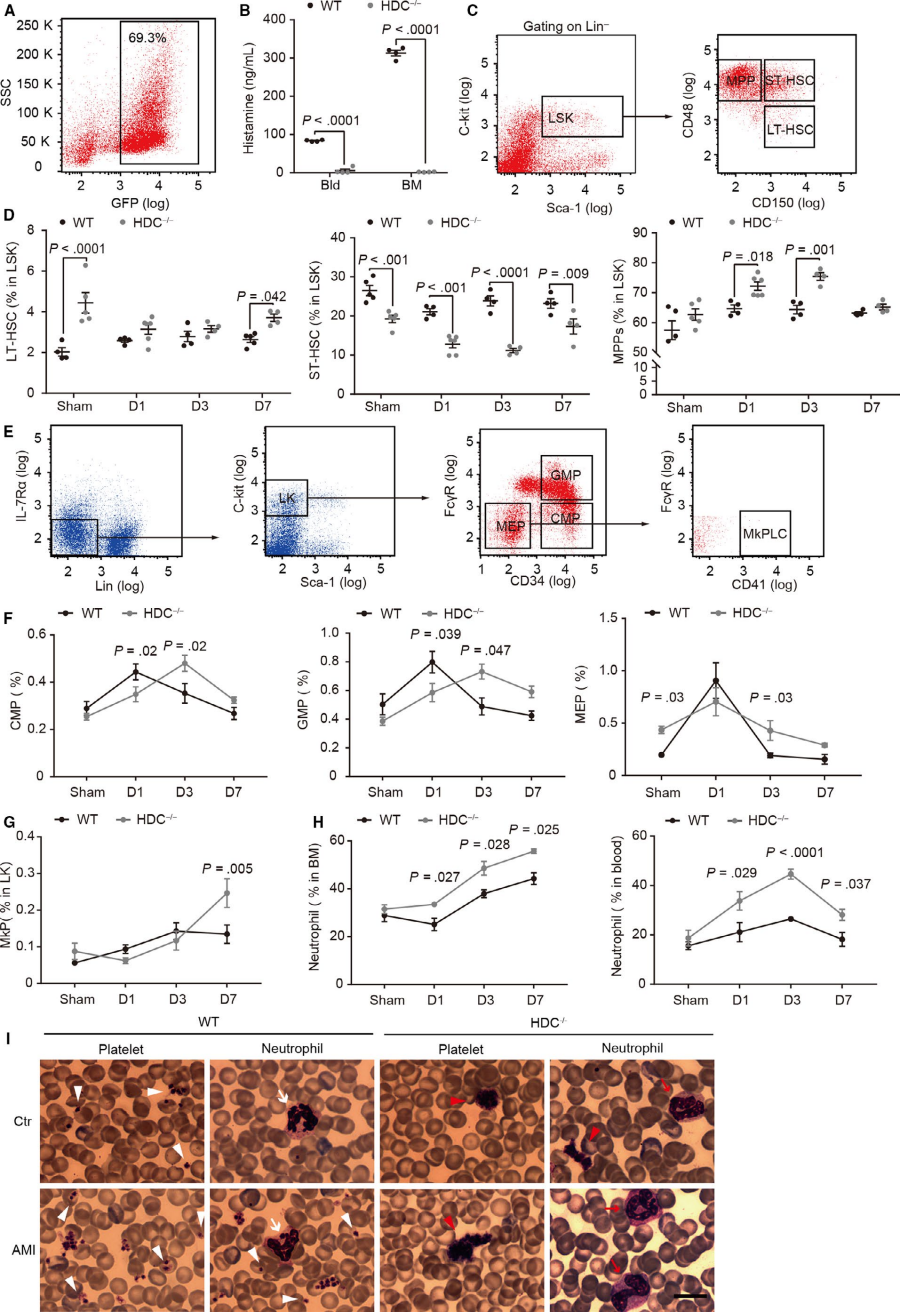
Histamine ablation facilitates thrombocytopoiesis and granulopoiesis after AMI. A, Fraction of HDC‐expressing cells in the bone marrow. B, Histamine level in the serum (Bld) and bone marrow (BM) lavage fluid. C‐G, Flow cytometry analysis of haematopoietic stem and progenitor cells (HSPCs) in the bone marrow. C, Gating strategy of long‐term HSCs (LT‐HSCs, CD48^−^CD150^+^Lin^−^Sca‐1^+^C‐kit^+^ [LSK]), short‐term HSCs (ST‐HSCs, CD48^+^CD150^+^LSK) and multipotent progenitors (MPPs, CD48^+^CD150^‐^LSK). D, Fraction of LT‐HSCs, ST‐HSCs and MPPs in LSK. E, Gating strategy of common myeloid progenitors (CMPs, CD34^+^FcγR^−^Lin^−^IL‐7Rα^−^Sca‐1^−^C‐kit^+^ [LK]), granulocyte‐macrophage progenitor (GMP, CD34^+^FcγR^+^LK), megakaryocyte‐erythroid progenitors (MEPs, CD34^−^FcγR^−^LK) and megakaryocyte progenitor cell‐like cells (MkPLCs, CD41^+^CD34^−^FcγR^−^LK). Fraction of (F) CMPs, GMPs, MPPs and (G) MkPLCs in LK. H, Fraction of neutrophils in the bone marrow and peripheral blood. I, Representative images of Wright‐Giemsa staining. Arrowheads show platelets (white, scattered platelets, and red, highly concentrated platelets). Arrows indicate neutrophils (white, mature neutrophils, and red, immature neutrophils). Bar: 20 μm. Graphs show mean ± SEM. *P*‐values were determined by one‐way ANOVA with Bonferroni‐Dunn correction

HDC‐expressing myeloid cells are the dominant cells in the bone marrow^12^ and maintain a high level of histamine in the bone marrow cavity (Figure 2A,B), suggesting that all the haematopoietic stem cells (HSCs) are immersed in a histamine‐rich environment. Histamine deficiency in HSCs niche results in the loss of HSCs quiescence and enhanced myeloid proliferation.^22^ Consistent with previous observations,^22^ we found that histamine deficiency activates long‐term HSCs (LT‐HSCs, identified as CD48^−^CD150^+^Lin^−^Sca‐1^+^C‐kit^+^[LSK]) and short‐term HSCs (ST‐HSCs, CD48^+^CD150^+^LSK) to quickly enter differentiation cycle, resulting in a significant increase in multipotent progenitors (MPPs, CD48^+^CD150^−^LSK) in response to infarct‐related myeloid demand (Figure 2C,D).

Together with HSCs consumption and MPPs increase, neutrophil precursors (including common myeloid progenitors [CMPs, CD34^+^FcγR^−^Lin^−^IL‐7Rα^−^Sca‐1^−^C‐kit^+^{LK}] and granulocyte‐macrophage progenitors [GMPs, CD34^+^FcγR^+^LK]) and platelet precursors (including megakaryocyte‐erythroid progenitors [MEPs, CD34^−^FcγR^−^LK] and megakaryocyte progenitor cell‐like cells [MkPLCs, CD41^+^CD34^−^FcγR^−^LK]) were significantly increased in HDC^−/−^ mice at D3/7 post‐AMI (Figure 2E‐G). This suggests a robust tendency to differentiate to neutrophils and platelets in HDC^−/−^ HSCs. We confirmed this speculation by analysis of CD11b^+^Ly6G^+^ neutrophils from the bone marrow and the peripheral blood, colony‐forming unit assay, and blood cell count (Figures 1G,H and 2H, and Figure S2A,B).

As histamine deficiency can exert its effects on HSCs, the most primitive precursors of blood cells, we hypothesized that their HDC^−/−^ descendants might exhibit abnormalities in morphological and/or functional characteristics. Indeed, we found that HDC^−/−^ platelets were highly aggregated on blood film, while WT platelets were scattered independently (Figure 2I). Furthermore, immature neutrophils with band nuclei were seen in the peripheral blood of HDC^−/−^ mice post‐AMI but not in WT mice (Figure 2I). These findings, which are suggestive of a lineage differentiation bias of HDC^−/−^ HSCs and morphological changes in HDC^−/−^ platelets and neutrophils, led us to further examine the impact of histamine on platelet and neutrophil function.

### 3.3 Activation and aggregation are increased in HDC^−/−^ platelets

Arterial thrombus formation is a complicated dynamic process which includes multiple synergetic and sequential steps. Among these steps, platelet adhesion and aggregation are crucial to the initiation and formation of arterial thrombus.^23^ To identify whether HDC^−/−^ platelets possess any functional changes that might contribute to microthrombosis, we carried out in vitro experiments on washed platelets.

**Figure 3:**
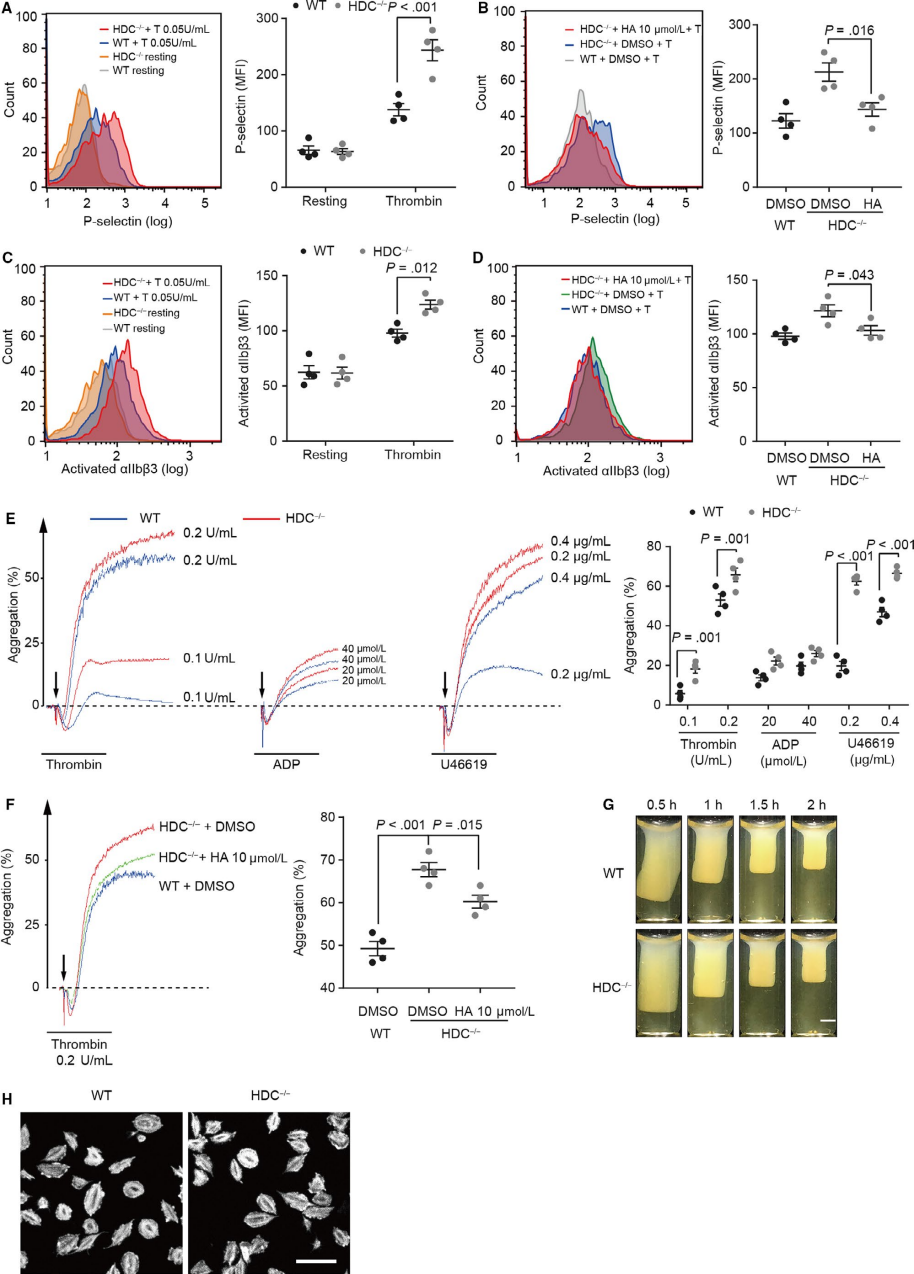
Activation and aggregation are increased in HDC^−/−^ Platelets. A‐D, Flow cytometry analysis of platelet activation. A, P‐selectin expression levels in resting platelets and thrombin (T, 0.05 U/mL)‐activated platelets. B, P‐selectin expression levels in HDC^−/−^ platelets incubated with thrombin (0.05 U/mL) after incubation with histamine (HA, 10 μmol/L) or DMSO. C, Activated αIIbβ3 levels in resting platelets and thrombin (T, 0.05 U/mL)‐activated platelets. D, Activated αIIbβ3 levels in HDC^−/−^ platelets incubated with thrombin (0.05 U/mL) after incubation with histamine (HA, 10 μmol/L) or DMSO. E, F, Aggregation curves of platelets. E, Addition of thrombin (high dose 0.2 U/mL, low dose 0.1 U/mL), ADP (40 μmol/L, 20 μmol/L), and U46619 (0.4 μg/mL, 0.2 μg/mL) in platelets. F, Thrombin (0.2 U/mL) induced aggregation after incubation with histamine (HA, 10 μmol/L) or DMSO in HDC^−/−^ platelets or DMSO in WT platelets. G, Thrombin‐induced clot retraction. Bar: 2 mm. H, Representative images of platelet spreading on fibrinogen‐coated slides. Bar: 10 μm. Graphs show mean ± SEM. *P*‐values were determined by one‐way ANOVA with Bonferroni‐Dunn correction

Platelet activation induces α‐granules to release numerous factors which mediate thrombus formation, such as P‐selectin. Thus, we measured thrombin‐induced α‐degranulation by determining P‐selectin expression levels on platelets. We found an increased P‐selectin expression level in thrombin‐stimulated HDC^−/−^ platelets (Figure 3A), indicating that HDC^−/−^ platelets exhibit a lower threshold to agonist and are more easily activated. Furthermore, the enhanced activation of HDC^−/−^ platelets can be down‐regulated by histamine administration (Figure 3B). Platelet activation also leads to αIIbβ3 activation, which is essential to mediate platelet aggregation and leucocyte‐platelet interaction. We found that histamine deficiency increased αIIbβ3 activation in response to thrombin (Figure 3C) and histamine administration down‐regulated this enhanced activation of αIIbβ3 in HDC^−/−^ platelets (Figure 3D). These results confirmed that histamine plays a crucial role in platelet activation. An enhancement in platelet activation will exponentially increase the number of participating platelets in thrombosis and even the subsequent aggregation process. Notably, HDC^−/−^ platelets manifested higher aggregation rates at both low dose and high dose of thrombin‐, adenosine diphosphate (ADP)‐ and U46619 (thromboxane A_2_ receptor agonist)‐induced aggregation (Figure 3E). Similar to its effect on platelet activation, histamine administration down‐regulated the elevated aggregation in HDC^−/−^ platelets (Figure 3F). These data indicate that histamine deficiency elicits platelet activation and aggregation.

Activated platelets undergo morphological transformation mediated by cytoskeleton to create filopodia and lamellipodia, following which they accomplish platelet contraction.^24^ However, no differences were detected in clot retraction, platelet spreading on fibrinogen‐coated slides or numbers of α‐granules, dense granules and mitochondria of HDC^−/−^ platelets, suggesting that histamine deficiency has no influence on the dynamics of cytoskeleton in platelets (Figure 3G,H and Figure S3A).

### 3.4 Histamine affects platelet function via histamine receptor‐mediated regulation of AKT phosphorylation

**Figure 4:**
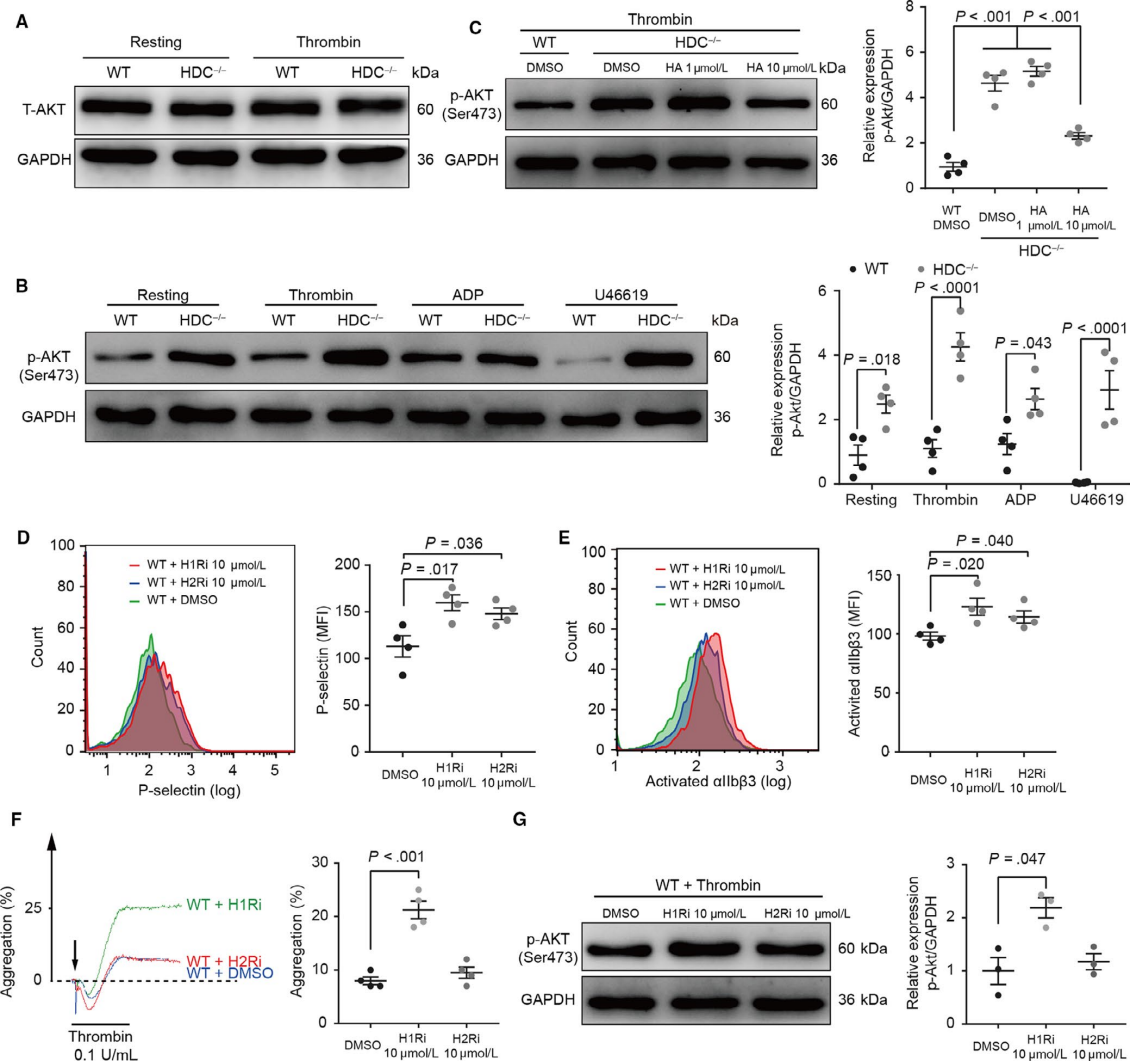
Histamine affects platelet function via histamine receptor‐mediated regulation of Akt phosphorylation. A‐C, Western blot analysis. Representative blots of (A) total Akt expression in resting and thrombin‐activated platelets, (B) phosphorylated Akt (Ser473) expression in platelets stimulated with thrombin (0.2 U/mL), ADP (40 μmol/L) and U46619 (0.4 μg/mL), and (C) phosphorylated Akt (Ser473) expression in platelets stimulated with thrombin (0.2 U/mL) after incubation with DMSO or histamine (HA). Flow cytometry analysis of (D) P‐selectin expression and (E) αIIbβ3 activation in thrombin (0.05 U/mL)‐stimulated WT platelets in the presence of selective antagonists for H1R (H1Ri, pyrilamine, red) and H2R (H2Ri, cimetidine, blue). DMSO served as a negative control (green). F, Thrombin (0.2 U/mL)‐induced aggregation after incubation with H1Ri, H2Ri or DMSO in WT platelets. G, Representative western blots of phosphorylated Akt (Ser473) expression in WT platelets stimulated with thrombin (0.2 U/mL) after incubation with DMSO, H1Ri or H2Ri. Graphs show mean ± SEM. *P*‐values were determined by one‐way ANOVA with Bonferroni‐Dunn correction

We next investigated the underlying intracellular signals involved in the relationship between histamine deficiency and increased platelet activation and aggregation. Given that Akt has been documented to play critical roles in platelet activation induced by αIIbβ3, GPIb‐IX‐V and G protein‐coupled receptors (GPCRs),^25^, ^26^ we investigated the effect of histamine deficiency on the Akt pathway. Total Akt expression remained unaffected upon activation, and histamine deficiency had no effect on its expression either (Figure 4A). However, enhanced Akt phosphorylation was found in HDC^−/−^ platelets in both the resting state and activated by thrombin, ADP and U46619 state (Figure 4B). Moreover, histamine administration down‐regulated the elevated Akt phosphorylation in thrombin‐induced aggregation of HDC^−/−^ platelets (Figure 4C). These data suggest that histamine regulates platelet activation and aggregation via an Akt‐dependent pathway.

Due to the fact that histamine functions by binding to its receptors (HRs), we hypothesized that the enhanced platelet activation and aggregation induced by histamine deficiency might be due to the lack of histamine/HRs axis‐mediated signals. Among four known HRs, we found that H1R and H2R were highly expressed on platelets (Figure S3B). To determine whether histamine exerts effects on platelets via these receptors, we carried out in vitro experiments on WT platelets pre‐treated with HR inhibitors. Blocking of histamine/HRs‐mediated intracellular signals by pyrilamine and cimetidine elevated P‐selectin expression level and αIIbβ3 activation on WT platelets in response to thrombin (Figure 4D). Moreover, inhibition of histamine/H1R‐mediated intracellular signals increased platelet aggregation rate and Akt phosphorylation (Figure 4F,G). Taken together, these data indicate that histamine takes part in the negative feedback regulation of platelet activation and aggregation involving, at least in part, H1R‐ and H2R‐dependent signals and the Akt pathway.

### 3.5 HDC^−/−^ platelets increase neutrophil‐platelet interactions

**Figure 5:**
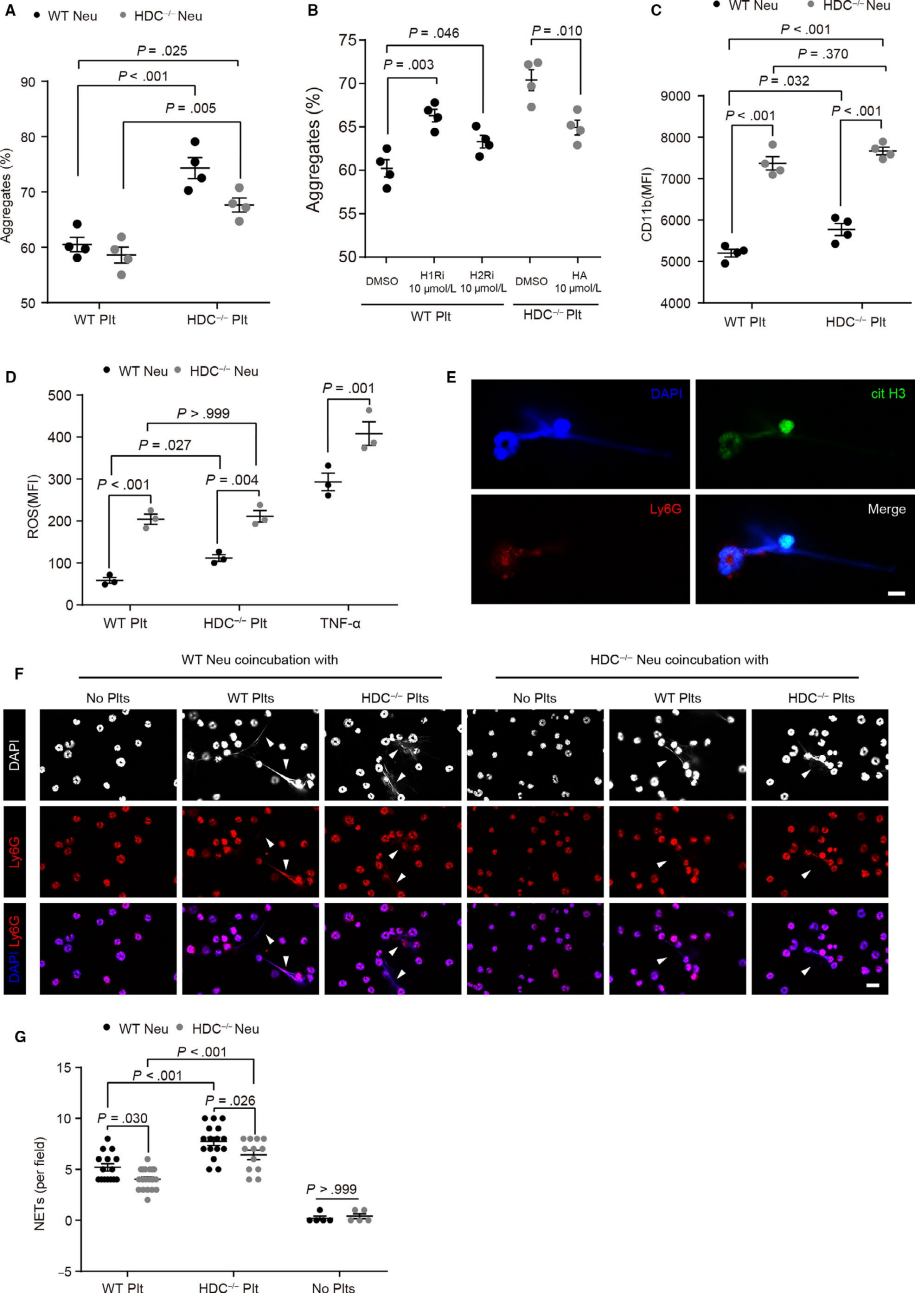
HDC^−/−^ platelets increase neutrophil‐platelet interactions. A‐F, Coincubation experiments. Washed platelets were pre‐treated with thrombin (0.05 U/mL) and neutrophil‐platelet interactions were analysed. A‐D, Flow cytometry analysis of (A) the formation of neutrophil‐platelet aggregates between WT/HDC^−/−^ platelets and WT/HDC^−/−^ neutrophils, (B) the formation of neutrophil‐platelet aggregates formation between WT neutrophils and WT platelets in the presence of pyrilamine (H1Ri, 10 μmol/L) or H2Ri (cimetidine, 10 μmol/L), or HDC^−/−^ platelets in the presence of histamine (HA, 10 μmol/L). C, CD11b expression on neutrophils, D, neutrophil intracellular formation of reactive oxygen species (ROS). TNFα (10 μmol/L)‐activated neutrophils served as a positive control in D. E‐G, Neutrophil extracellular traps (NETs) formation assay. E, Representative images of NETs. DAPI (nuclei, blue), Ly6G (neutrophil, red), citrullinated histone H3 (citH3, green). Bar: 5 μm. F, NETs formation induced by thrombin‐stimulated platelets. Upper row (DAPI, nuclei, white), middle row (Ly6G, neutrophil, red), and bottom row (merged images of DAPI in blue and Ly6G in red). Arrowheads indicate NETs. Bar: 10 μm. G, Quantitative analysis of NETs formation. Graphs show mean ± SEM. *P*‐values were determined by two‐way ANOVA with Tukey multiple comparisons or one‐way ANOVA with Bonferroni‐Dunn correction (B)

Binding to activated platelets is crucial for the recruitment of circulating neutrophils and the initiation of inflammatory responses.^7^, ^8^ Histamine deficiency led to elevated P‐selectin expression levels, αIIbβ3 activation and platelet aggregation, which led us to investigate whether these functional alterations facilitate neutrophil‐platelet interactions. We found that the formation of neutrophil‐platelet aggregates with either WT neutrophils or HDC^−/−^ neutrophils was increased in incubation with thrombin pre‐stimulated HDC^−/−^ platelets when compared to WT controls (Figure 5A). When we focused on neutrophils, HDC^−/−^ neutrophils were shown to reduce the formation of neutrophil‐platelet aggregates, suggesting that histamine deficiency may lead to changes in neutrophil function. However, the formation of neutrophil‐platelet aggregates in the HDC^−/−^ platelets/HDC^−/−^ neutrophils group, which mimics the histamine‐deficient environment in HDC^−/−^ mice, still exceeded that of the WT platelet/WT neutrophil group. Moreover, blocking of histamine/HRs‐mediated intracellular signals in WT platelets by pyrilamine and cimetidine increased the formation of neutrophil‐platelet aggregates, while histamine pre‐incubation in HDC^−/−^ platelets down‐regulated the enhanced neutrophil‐platelet aggregates formation (Figure 5B).

Leucocyte integrin CD11b is a marker of neutrophil activation and crucial for leucocyte recruitment to endothelium.^27^ Moreover, CD11b binding with GPIbα is indispensable in arterial thrombus formation.^7^ We found that HDC^−/−^ platelets led to an increase in CD11b expression in WT neutrophils, while HDC^−/−^ neutrophils exhibited high CD11b expression levels prior coincubation with platelets (Figure 5C), suggesting an abnormal expression of integrin on HDC^−/−^ neutrophils. Furthermore, HDC^−/−^ platelets led to an increased production of reactive oxygen species (ROS) in WT neutrophils, but had no effect on HDC^−/−^ neutrophils, which already had high levels of ROS production (Figure 5D). High ROS levels help neutrophils defend against extracellular stimuli, but also lead to excessive oxidative injury.^28^

Other than oxidative burst, NETs is another cellular effect that matters during neutrophil activation.^29^ Activated platelets were shown to induce the release of extracellular nucleosomes from neutrophils, which is reflected by the positive stain for citrullinated histone 3 (cit H3), a marker for neutrophil priming towards NETosis (Figure 5E). HDC^−/−^ platelets increased the formation of NETs with either WT neutrophils or HDC^−/−^ neutrophils when compared to WT controls, while HDC^−/−^ neutrophils showed a decrease in NETs formation irrelevant of platelet coincubation (Figure 5F,G). Notably, the formation of NETs in the HDC^−/−^ platelets/HDC^−/−^ neutrophils group exceeded that of the WT platelet/WT neutrophil group. In summary, HDC^−/−^ platelets increased both neutrophil activation and neutrophil‐platelet interactions while HDC^−/−^ neutrophils exhibit abnormalities in integrin expression levels and the activation process.

### 3.6 HDC^−/−^ platelets promote neutrophil recruitment

**Figure 6:**
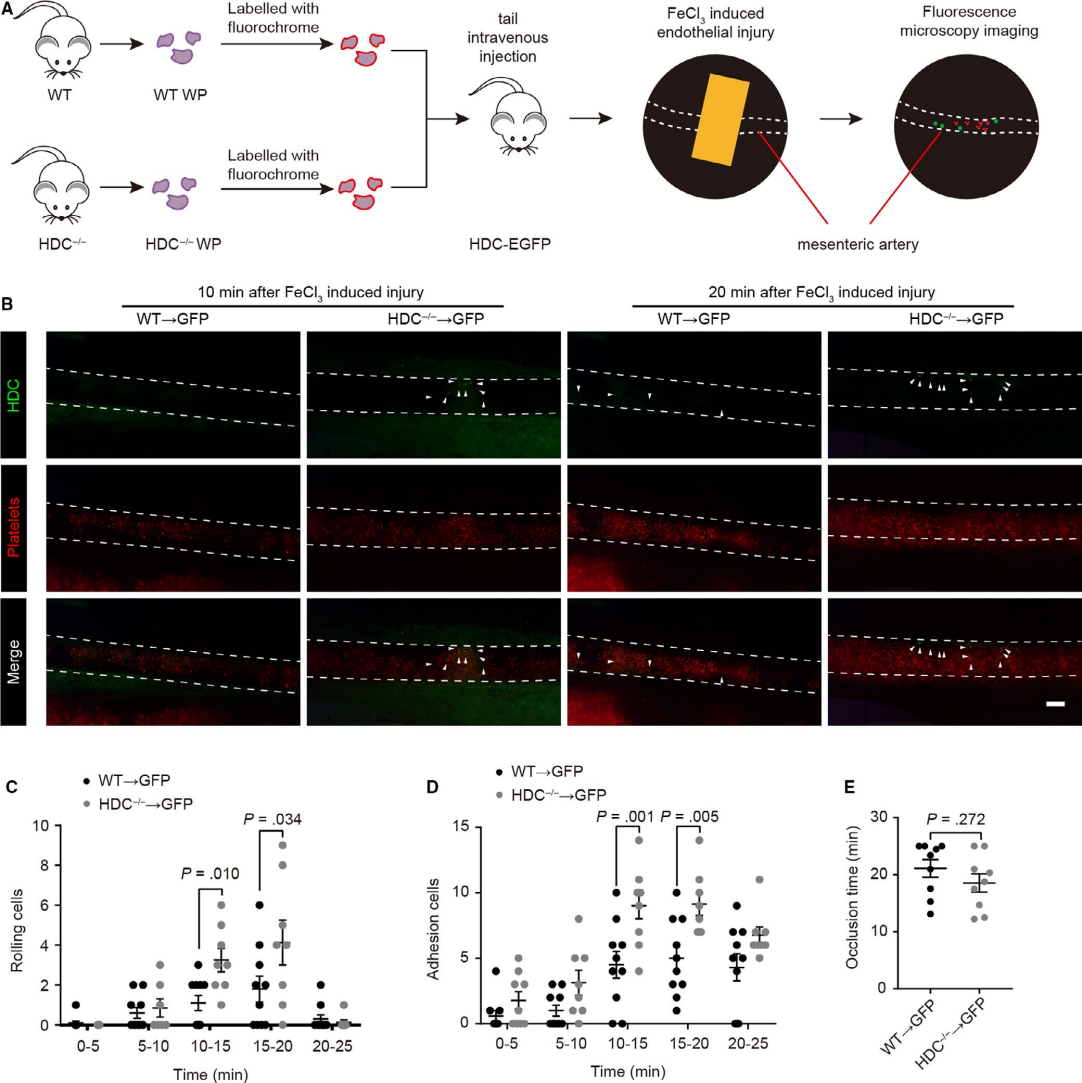
HDC^−/−^ platelets promote neutrophil recruitment. A‐F, Intravital microscopy of FeCl_3_‐induced thrombosis in mouse mesenteric arterioles. Washed platelets were isolated and labelled with DiD. A total of 10^7^ platelets were then injected into HDC‐EGFP (neutrophil reporter) recipient mice through the tail vein. A, Illustration of intravital microscopy. B, Representative intravital microscopy images of platelets (red) and neutrophils (green). Arrowheads show recruited HDC‐expressing neutrophils. Bar: 100 μm. C, Neutrophil rolling and D, adhesion in mesenteric arterioles. E, Time until mesenteric arterioles were occluded by thrombus. Graphs show mean ± SEM. *P*‐values were determined by one‐way ANOVA with Bonferroni‐Dunn correction

Having uncovered that histamine deficiency results in platelet and neutrophil dysfunction and enhances neutrophil‐platelet interactions in vitro, we investigated whether histamine deficiency influences neutrophil‐platelet interactions and thrombosis in vivo. Platelets isolated from WT or HDC^−/−^ donor mice were labelled with DiD and then injected through the tail vein into HDC‐EGFP mice, in which most neutrophils and fractional monocytes express EGFP.^14^, ^22^ Intravital microscopy was performed in a model of FeCl_3_‐induced endothelial injury of the mesenteric arterioles (Figure 6A). During thrombosis, platelets were first activated and rapidly aggregated to form thrombus (Figure 6B and Videos S2 and S3). Subsequently, HDC‐expressing neutrophils collided and bonded with these platelets, resulting in neutrophil rolling and adhesion to the thrombus or injured endothelium (Figure 6B and Videos 2,3). In accordance with the results of our in vitro studies, HDC^−/−^ platelets increased neutrophil rolling and adhesion to the injured endothelium of mesenteric arterioles, especially at 10‐20 minute after FeCl_3_ infiltration (Figure 6B‐D and Videos S2 and S3). Taken together, these results indicate that HDC^−/−^ platelets increase neutrophil‐platelet interactions and promote neutrophil recruitment at the sites of endothelial injury.

In addition, the mean occlusion time of the mesenteric arterioles remained unaffected (Figure 6E), indicating that injection of HDC^−/−^ platelets failed to result in accelerated occlusion. Given that only a fraction of platelets in these chimeric mice were hyperactive, the majority of them still exhibited a regular phenotype; therefore, the enhanced neutrophil‐platelet interactions and the subsequently accelerated thrombosis might not be as evident.

## 4 DISCUSSION

**Figure 7:**
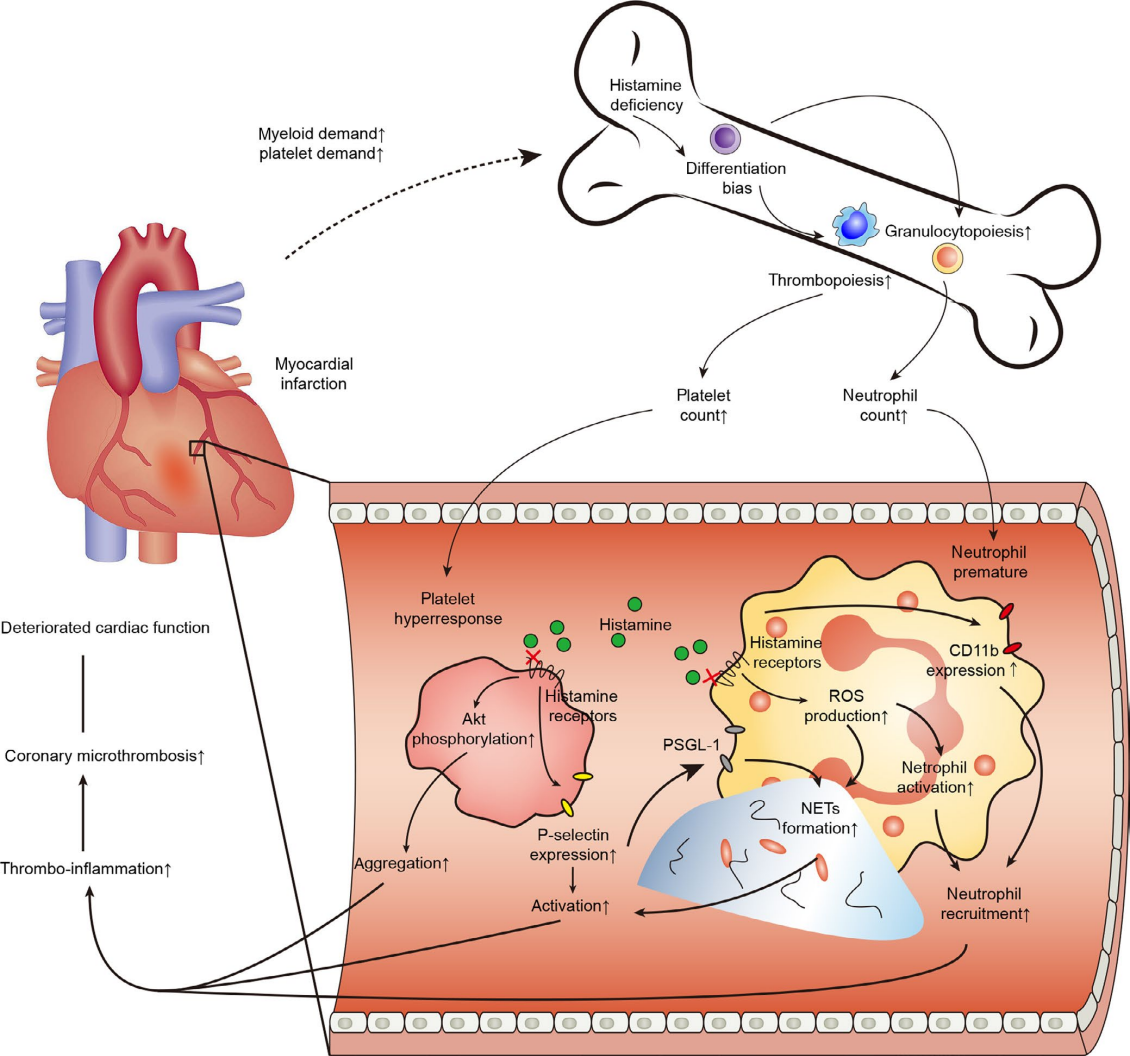
Graphical illustration. Myocardial infarction triggers acute inflammatory responses, and the circulating platelets and neutrophils are activated and respond rapidly in the ischemic region. Along with the consumption of platelets and neutrophils, HSCs in the bone marrow are subsequently activated to generate more descendants according to the myeloid and platelet demands. Histamine ablation facilitates thrombocytopoiesis and granulopoiesis after AMI, leading to increased number of circulating platelets and neutrophils. Absence of histamine/HRs‐mediated intracellular signals elevates P‐selectin expression level and αIIbβ3 activation on platelets by enhancing Akt phosphorylation. HDC^−/−^ platelets promotes neutrophil recruitment, increases the formation of neutrophil–platelet aggregates, and elevates CD11b expression and NETs formation in neutrophils. Activated platelets excite neutrophils, which, in return, reinforces platelet activation. Taken together, changes in HDC^−/−^ HSCs differentiation bias and functional alterations in their descendants contribute to the increased neutrophil‐platelet interactions and enhanced thrombo‐inflammation together, leading to increased number of coronary microthrombi and deteriorated cardiac function

In this study, we have demonstrated a previously unknown mechanism by which neutrophil‐derived histamine exerts protective effects against excessive arterial thrombosis post‐AMI. Our results suggest that histamine deficiency triggers enhanced neutrophil‐platelet interactions and leads to increased microthrombosis. Our study also provides evidence that a histamine‐deficient environment generates dysfunctional neutrophils and platelets, which further contribute to microthrombosis (illustrated in Figure 7).

Neutrophils have been recently identified in the coronary artery thrombi of AMI patients.^5^, ^6^ Neutrophils are a cluster of HDC‐expressing cells, responsible for histamine homeostasis in the bone marrow and serum.^14^, ^30^ Via surface expression of CD11b and P‐selection glycoprotein ligand‐1 (PSGL‐1), neutrophils interact with platelets to contribute to platelet activation and aggregation, potentiate thrombosis, and initiate inflammation.^7^, ^29^ Our findings demonstrate that histamine‐producing neutrophils directly engage in arterial thrombosis. In injury‐related inflammation, histamine‐producing neutrophils are rapidly recruited to the injured site at the endothelial surface. While it was impossible to visualize the release of histamine from neutrophils and their direct binding to platelets by intravital microscopy due to technical limitations, our data clearly demonstrate the recruitment of histamine‐producing neutrophils in arterial thrombi and their direct interactions with activated platelets. Moreover, we provide evidence that histamine deficiency increased coronary microthrombosis after AMI and induced robust infarct‐related inflammation in HDC^−/−^ mice, thereby contributing to cardiac function deterioration.

Neutrophil extracellular traps, containing decondensed chromatin and antimicrobial proteins, stimulate the intrinsic pathway of coagulation, increase platelet‐dependent thrombin generation, activate platelets and increase thrombi size.^11^, ^19^, ^31^, ^32^ Thus, activated neutrophils play a procoagulant role in arterial thrombosis. Activated platelets excite neutrophils, which, in return, reinforces platelet activation. This bidirectional interaction is further supported by up‐regulation of CD11b expression, ROS production and NETs formation, all of which can avail thromboinflammation.^33^, ^34^ However, HDC^−/−^ neutrophils are immature cells.^14^ Although HDC^−/−^ neutrophils possess procoagulant characteristics supported by increased CD11b expression and high ROS production, histamine deficiency may lead to functional alterations which may explain why HDC^−/−^ neutrophils reduce the formation of neutrophil‐platelet aggregates and NETs. Nevertheless, our findings suggest that HDC^−/−^ platelets increase the formation of neutrophil‐platelet aggregates, foster ROS production in vitro and facilitate neutrophil recruitment at sites of endothelial injury in vivo, which eventually promote thromboinflammation. Furthermore, given that neutrophil and platelet counts after AMI are much higher in HDC^−/−^ mice, we conclude that neutrophil‐platelet interactions in HDC^−/−^ mice are much more grievous.

P‐selectin and β2‐containing integrins are essential for neutrophil recruitment.^35^ The interaction of P‐selectin with PSGL‐1 increases calcium entry in platelets and triggers platelet αIIbβ3 activation, leading to platelet aggregation and leucocyte‐platelet aggregates.^36^, ^37^ Here, we show that histamine deficiency increases P‐selection expression, αIIbβ3 activation, platelet aggregation and the formation of neutrophil‐platelet aggregates. Previous studies have indicated that histamine reduces GPIbα‐mediated adhesion of platelets to inflamed endothelium, which supports our findings.^38^ Furthermore, the PI3K/Akt pathway plays a critical role in platelet activation induced by various receptors, such as αIIbβ3 and GPIb‐IX‐V.^25^, ^26^ In gastric parietal cell, modulation of Akt phosphorylation is involved in histaminergic stimulation‐mediated gastric acid secretion.^39^ Our results suggest that Akt phosphorylation in HDC^−/−^ platelets is enhanced in both resting and activated platelets. Taken together, our data demonstrate that histamine deficiency in platelets intervenes in platelet α‐degranulation and activation, resulting in increased Akt phosphorylation and platelets aggregation.

Other than neutrophils, monocytes/macrophages are also HDC‐expressing cells.^12^, ^14^ Furthermore, an increased number of monocyte‐platelet aggregates were found in AMI patients.^40^ Activated platelets interact with monocytes directly, leading to the increased expression of CD40, CD11b and PSGL‐1 on monocytes. This results in the formation of monocyte‐platelet aggregates in the inflammatory area and promotes monocyte recruitment.^41^, ^42^ Moreover, histamine deficiency impairs macrophage infiltration and suppresses the healing process post‐AMI, which interferences with the clearance of debris and tissue repair.^12^ Histamine deficiency also promotes the apoptosis of cardiomyocytes after AMI^12^. As such, we suggest that histamine deficiency might also exert influences on other cells, such as endothelial cells. Further studies are required to verify whether histamine deficiency alters endothelial cell functions which may contribute to mediating cardiac injury.

The four HRs establish the basis of the versatile cellular effects exerted by histamine, such as neural information transmission, cardiac remodelling and allergic inflammation.^43^, ^44^, ^45^ Histamine administration rescues cardiac function deterioration and heart dilation after AMI in HDC^−/−^ mice and, this protection can be aborted by the administration of H1R and H2R inhibitors.^46^ Previous studies have reported the detection of histamine and HDC in human platelets.^47^ However, our findings suggest that platelets do not carry out endogenous histamine synthesis per se, as confirmed in HDC‐EGFP mice. The expression of green fluorescent protein in HDC‐EGFP mice is driven by the HDC promoter and therefore reflects the transcription and expression status of the *Hdc* gene.^22^ HDC/EGFP is last detected in MEPs throughout the megakaryocyte lineage.^22^ The sharp drop in HDC expression from MEPs to megakaryocytes indicates loss of multipotency, accompanied by differentiation and maturation. Platelet‐holding histamine seems more likely to be imported into the cell from the extracellular matrix, in which histamine primarily comes from CD11b^+^ myeloid cells.

Neutrophil‐derived histamine is primarily required for stem cell quiescence and myeloid proliferation in a histamine/H2R‐dependent manner.^22^ Our findings further proved that HSCs are immersed in a histamine‐rich environment in the bone marrow. In response to inflammatory demand released from ischaemic cardiomyocytes or injured coronary arteries and the consumption of platelets and neutrophils, proliferation and mobilization of platelets and neutrophils are abnormally enhanced in the HDC^−/−^ environment. Moreover, our findings suggest that changes in HDC^−/−^ HSCs differentiation bias and functional alterations in their descendants contribute to the enhanced coronary microthrombosis together. Further studies are needed to clarify the underlying mechanisms of these histamine deficiency‐induced functional alterations.

The increase in leucocyte‐platelet aggregates is independently associated with the no‐reflow phenomenon after percutaneous coronary intervention (PCI) and may predict the no‐reflow phenomenon in AMI patients.^48^ Depletion of platelets and restraint of neutrophils recruitment or NETosis would benefit thromboinflammatory disease without affecting haemostasis.^7^, ^11^ As such, when taking into account of the significance of neutrophil‐platelet interactions in thromboinflammatory diseases, a deeper understanding of the mechanisms regulating these interactions may lead to novel therapeutic routes. Notably, this study has been focused on a mice model only, in which artificial surgery was used to mimic AMI, as myocardial infarction cannot spontaneously occur in mice. Notwithstanding its limitation, this study does suggest that histamine plays a protective role against aggravating thromboinflammation.

## CONFLICT OF INTEREST

The authors confirm that there are no conflicts of interest.

## AUTHOR CONTRIBUTION

HL, CD, XY and JG were involved in study design, experiments performance (HL), data analysis and manuscript preparation. CT, XZ and MA helped with experiments performance and data processing. WZ helped with animal caring. SD helped with histological examinations. CD, XY and JG helped with manuscript revision. All authors discussed the results and commented on the manuscript.

## Data Availability

The processed and normalized datasets supporting the conclusions of this article are included within the article (Appendix S1). Raw data used during the current study are available from the corresponding author upon reasonable request.
