# Supplementary material for: Histamine deficiency facilitates coronary microthrombosis after myocardial infarction by increasing neutrophil‐platelet interactions
Source: J Cell Mol Med. 2020 Feb 16;24(6):3504–20. doi: 10.1111/jcmm.15037 (PMC7131923; doi:10.1111/jcmm.15037)
Supplement: Supplementary file 1 [file JCMM-24-3504-s001.docx]

**Supplementary files**

**Supplementary Methods**

**Table S1 Reagents**

| Reagents | Company | Cat No. | Concentrations |
| --- | --- | --- | --- |
| Histamine (HA) | Sigma-Aldrich | H7250 | 4 mg/kg (AMI surgery)  10 μM (platelet assays) |
| Pyrilamine (H1Ri) | Sigma-Aldrich | P5514 | 10 mg/kg (AMI surgery)  10 μM (platelet assays) |
| Cimetidine (H2Ri) | Sigma-Aldrich | C4522 | 10 mg/kg (AMI surgery)  10 μM (platelet assays) |
| Thrombin (T) | Enzyme research Laboratories | HT1002a | 0.5 U/mL (clot retraction)  0.2 U/mL, 0.1 U/mL (aggregation)  0.05 U/mL (activation) |
| ADP | Ameresco | 0160 | 40 μM, 20 μM (aggregation) |
| U46619 | Sigma-Aldrich | D8174 | 0.4 μg/mL, 0.2 μg/mL (aggregation) |
| PGE_1_ | Sigma-Aldrich | P5515 | 0.1 μg/mL (platelet preparation) |
| Apyrase | Sigma-Aldrich | A6535 | 1 U/mL (platelet preparation) |

**Table S2 Fluorochrome-conjugated antibodies used in flow cytometry analysis**

| Experiments | Antibodies | Cat No. | Company |
| --- | --- | --- | --- |
| HSCs analysis | hematopoietic lineage cocktail | 133302 | Biolegend |
|  | Sca-1 | 108114 | Biolegend |
|  | C-kit | 135119 | Biolegend |
|  | CD34 | 152208 | Biolegend |
|  | CD48 | 47-0481-82 | eBioscience |
|  | CD150 | 12-1502-82 | eBioscience |
|  | IL-7Rα | 135040 | Biolegend |
|  | FcγR | 101308 | Biolegend |
|  | CD41 | 133914 | Biolegend |
|  | CD11b | 17-0112-82 | eBioscience |
|  | Ly6G | 127618 | Biolegend |
| Platelet activation | CD41 | 133916 | Biolegend |
|  | P-selectin (CD62P) | 148304 | Biolegend |
|  | Activated αIIbβ3 (JON/A) | M023-2 | Emfret Analytics |
| Neutrophil-platelet aggregates formation | CD41 | 133916 | Biolegend |
|  | Ly-6G | 551461 | BD Biosciences |
|  | CD11b | 17-0112-82 | eBioscience |

**Enzyme-linked immunosorbent assay (ELISA)**

Histamine concentration in serum and bone marrow lavage fluid (100 μL phosphate buffered saline (PBS) for one femur cavity flush) were determined using the Histamine ELISA Kit (Eagle Biosciences, Cat No.HIS31-K01) according to the manufacturer’s instructions and detected using a SpectraMax M5 microplate reader (Molecular Devices).

**Colony-forming unit assay**

Bone marrow cells were harvested, erythrocytes were lysed with FACS lysing buffer (BD Biosciences, Cat No.349202) and the resuspended cells were labelled with diluted (1:200) antibodies. Antibody information is available in Table 2. Hematopoietic stem cells (HSCs) labelled as Lin^-^Sca-1^+^C-kit^+^CD48^-^CD150^+^ were isolated by MoFlowXDP cell sorter (Dako). Colony-forming unit assays were performed using MethoCult GF M3434 medium (Stem Cell Technologies) and colonies were scored after 8-12 d of incubation according to the manufacturer’s instructions.

**Platelet functional assays**

Platelet preparation, aggregation, spreading, and clot retraction were processed as previously described [1] and detailed as follows:

**Platelet preparation**

Blood obtained by terminal inferior vena cava phlebotomy were collected into syringes containing 100 μL/mL White anticoagulant (2.94% sodium citrate, 136 mM glucose), 0.1 μg/mL PGE_1_ (Sigma-Aldrich, Cat No.P5515), and 1 U/mL apyrase (Sigma-Aldrich, Cat No.A6535) [1]. Washed platelets were prepared from platelet-rich plasma by differential centrifugation and suspended in modified Tyrode’s buffer (12 mM NaHCO_3_, 138 mM NaCl, 5.5 mM glucose, 2.9 mM KCl, 2 mM MgCl_2_, 0.42 mM NaH_2_PO_4_, 10 mM HEPES, pH 7.4) [1]. Washed platelets were adjusted to the concentration required for respective experiments. Platelet counts were obtained using a resistance particle counter (Coulter Z2).

**Platelet aggregation**

Aggregation was started by adding thrombin, ADP and U46619 under continuous stirring at 1000 rpm at 37 °C and measured in a two-channel-lumiaggregometer (Chrono-Log 490-2D) using 300 μL washed platelets (10^6^/mL) [1]. Percentage of maximal platelet aggregation was analysed 5 min after addition of the agonist using the Aggro-link software (Chrono-Log). Histamine (HA, 10 μM), pyrilamine (H1Ri, 10 μM) and cimetidine (H2Ri, 10 μM) were incubated with the platelets for 3 min before stimulation. Detailed information regarding reagents is available in Table S1.

**Platelet spreading**

Washed platelets (2×10^7^/mL) were allowed to spread for 2 h on fibrinogen (Sigma-Aldrich, Cat No.F3879)-coated slides. Non-adherent platelets were removed after gently washing with PBS and spreading platelets were fixed with 4% formaldehyde, permeabilized with 0.5% Triton-X-100, blocked with 1% BSA and stained with fluorescein-conjugated phalloidin (Invitrogen, Cat No.F432). Images were recorded with a Leica SP8 confocal microscope and processed with the LASX software.

**Clot retraction**

Washed platelets (1×10^8^/mL) were added to fresh plasma at the volume ratio of 1:3 and dispensed in 0.4 mL aliquots into cuvettes. Clot retraction was induced by stimulation with thrombin (0.5 U/mL) at 37°C and monitored by taking photographs at indicated time points using a digital camera. Clot size was quantified from photographs using Image-Pro Plus 6.0 software (Media Cybernetics).

**Western Blotting**

After stimulation, platelets were immediately lysed on ice using RIPA lysis buffer (Beyotime, Cat No.P0013K) supplemented with a cocktail of protease, phosphatase and PMSF inhibitors (Biotechwell, Cat No.WB0122). Protein concentrations were determined using the BCA reagent kit (Beyotime, Cat No.P0012) according to the manufacturer’s instructions. Equal amounts of protein samples were separated by gel electrophoresis (SDS-PAGE) and then transferred onto polyvinylidene fluoride (PVDF) membranes (Millipore). Membranes were blocked using 5% bull serum albumin (Biotechwell, Cat No.WH1044) in Tris-buffered saline with Tween (TBST) for 30 min , incubated with primary antibodies (1:1000) at 4 °C overnight, washed with TBST afterwards, and then incubated with horseradish peroxidase-conjugated secondary antibodies for 1 h at room temperature. Rabbit anti-mouse antibodies against Akt (Cat No.4691) and phospho-Akt Ser473 (Cat No.4060) were purchased from Cell Signaling Technology. Mouse anti-mouse GAPDH antibody (Cat No.ab8245) was purchased from Abcam. Enzymatic activity was detected with an enhanced chemiluminescence kit (Millipore) according to the manufacturer’s instructions and recorded using a Bio-Rad imaging system. Densitometric assessment of the blots was performed using the Image-Pro Plus 6.0 software (Media Cybernetics).

**Transmission** **electron microscopy (TEM)**

Platelet sediments were fixed with 2.5% glutaraldehyde and then post-fixed with 1% osmium tetraoxide, dehydrated in a graded series of ethanol concentrations and embedded in Embed812 resin. The ultrathin sections were mounted on copper grids and then double-stained with uranyl acetate and lead citrate. Samples were examined and photographed with a FEI Tecnai T20 electron microscope. Dense granules, α-granules and mitochondria were identified and counted manually per platelet.

**Quantitative real-time PCR (Q-PCR)**

Total RNA was extracted with Trizol reagent (Invitrogen, Cat No.15596018) and used for cDNA synthesis with random primers (Takara, Cat No.RR037A). Q-PCR was carried out by the SYBR Green PCR master Mix (Takara, Cat No.RR420A) according to the manufacturer’s instructions. Data were recorded by the real-time PCR systems (Bio-Rad) and processed with the Bio-Rad CFX software. The relative mRNA expression level was determined using the 2^-∆∆CT^ method [2] and Actin expression was used as endogenous control. Primers are listed as below and primer efficiency are showed in Figure S5B：

| Gene | Forward (5′-3′) | Reverse (5′-3′) |
| --- | --- | --- |
| Actin | TGCTTCTAGGCGGACTGTTAC | AACCAACTGCTGTCGCCTT |
| H1R | TGACGTCATGCAGACACAGC | CCGGGAGAAGGACTGTCGAT |
| H2R | TCTCCTTCCTCTCTATTCACCT | CATCCACCAGTCCATATACCTC |
| H3R | CTATGACCGATTCCTGTCAGTC | CGTGATGAGAAAGTACCAGTTG |
| H4R | GGACAGAAACCTTAGACATCGA | AAAACATGCAGATTCCACTTCC |

**Neutrophil-platelet interaction assays**

Neutrophil preparation, neutrophil-platelet aggregates formation and NETs formation in vitro were processed as previously described [3] and detailed as follows:

**Isolation of neutrophils**

Briefly, isolation of the neutrophils from bone marrow was carried out using a discontinuous isotonic Percoll gradient (52/64/72%, Yeasen, Cat No.40501ES60) and centrifuged at 1000×g for 30 min. Neutrophils were collected from the 64/72% interface, washed in PBS and then cultivated for 24 h in RPMI1640 medium (Gibco, Cat No.31870082) at 37 °C and 5% CO_2_. Neutrophil viability evaluated by trypan blue exclusion test was > 95%.

**Neutrophil-platelet aggregates formation and neutrophil activation *in vitro***

Washed platelets were treated with 0.05 U/mL thrombin for 20 min and labelled with fluorochrome-conjugated antibody to mouse CD41 (Biolegend, Cat No.133916). Neutrophils were labelled with fluorochrome-conjugated antibody to mouse Ly-6G (BD Biosciences, Cat No.551461). A total of 2x10^7^ platelets were co-incubated with 10^6^ neutrophils in a volume of 400 μL neutrophil adhesion medium (HBSS supplemented with 20 mM HEPES, 0.25% BSA, 0.1% glucose, 1.2 mM Ca^2+^, and 1.0 mM Mg^2+^). Analysis of neutrophil–platelet aggregates was carried out by flow cytometry. Neutrophils were identified as Ly6G^+^ cells, and aggregates expressed as the percentage of platelet (CD41)-positive neutrophils (Figure S3C). In a separate set of experiments, neutrophil activation upon platelet co-incubation was assessed with a fluorochrome-conjugated anti-mouse CD11b antibody (eBioscience, Cat No.17-0112-82). In another separate set of experiments, neutrophil intracellular ROS generation was determined using 10 μM 2’-7’-dichlorodihydrofluorescein diacetate (DCFH-DA, Beyotime, Cat No.S0033). Stimulation of neutrophils with TNFα (Peprotech, Cat No. 300-01A) served as positive control. Data were acquired using a FACS Canto flow cytometer (BD Biosciences) and processed with the FlowJo v10 software (Tree star).

**NETs formation *in vitro***

Platelets were treated with 0.05 U/mL thrombin for 20 min, washed afterwards and added to neutrophils and co-incubated for 1 h at 37 °C. After stimulation, the non-adherent cells were gently removed by washing. The adherent cells were then fixed with 4% paraformaldehyde, stained with fluorochrome-conjugated antibody to mouse Ly-6G (BD Biosciences, Cat No.551461) and DAPI to visualize NETs structure. Anti-mouse histone H3 (citrulline R2+R8+R17) antibody (Abcam, Cat No.ab5103) and corresponding secondary antibody (Abcam, Cat No.ab150077) were used for NETs formation identification. Images were acquired on a Leica SP8 confocal microscope and processed with the LASX software. Quantitative analysis was carried out by manual counting of NET-structures per visual field of 3-5 randomly defined ROIs per sample.

**References**

1. Chen X, Zhang Y, Wang Y, et al. PDK1 regulates platelet activation and arterial thrombosis. *Blood.* 2013;121(18):3718-3726. DOI:10.1182/blood-2012-10-461897
2. Livak K J and Schmittgen TD. Analysis of relative gene expression data using real-time quantitative PCR and the 2(-Delta Delta C(T)) Method. *Methods*. 2001;25(4):402-408. DOI:10.1006/meth.2001.1262
3. Pircher J, Czermak T, Ehrlich A, et al. Cathelicidins prime platelets to mediate arterial thrombosis and tissue inflammation. *Nat Commun*. 2018;9(1):1523. DOI:10.1038/s41467-018-03925-2

**Supplementary figures**

**
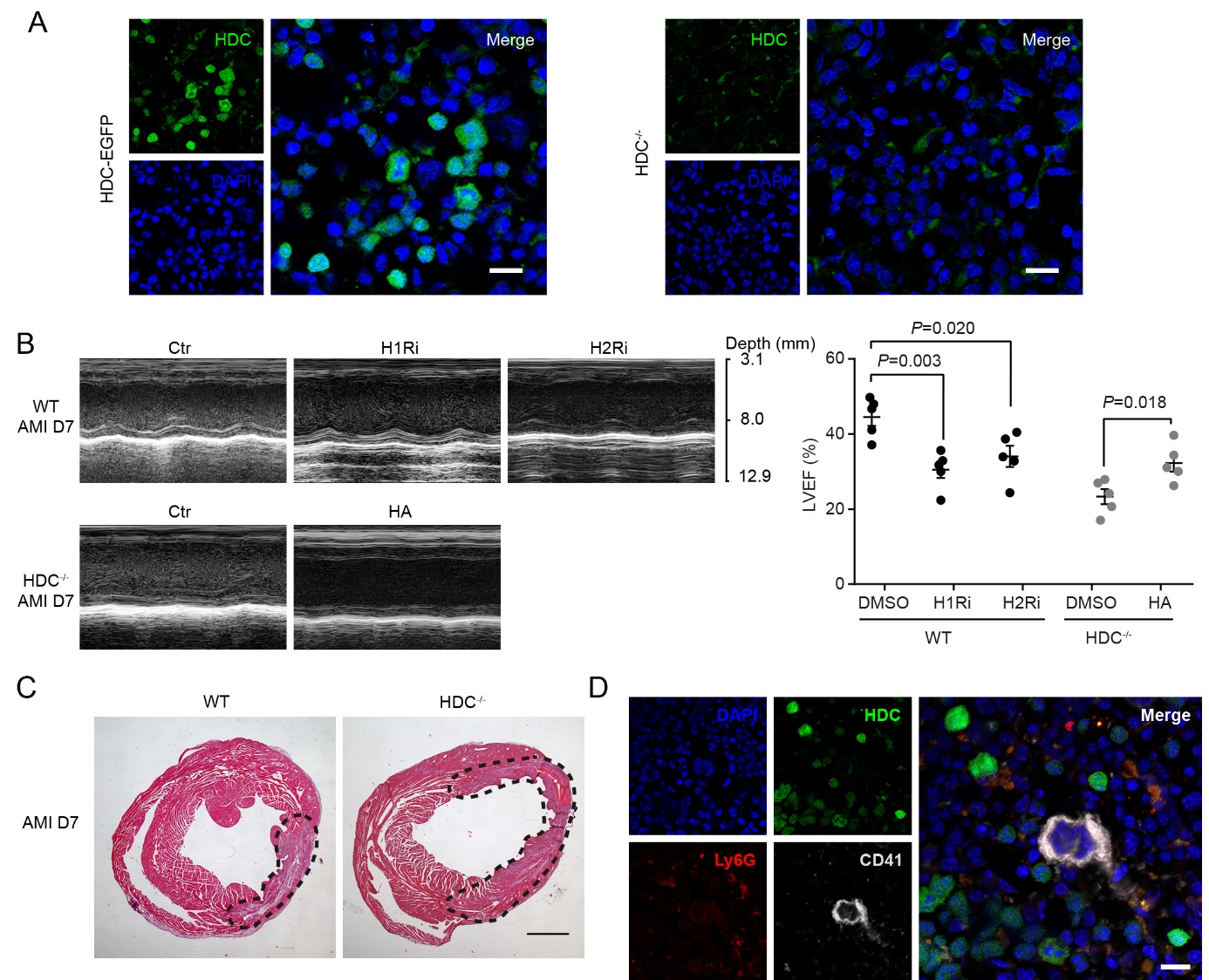
**

**Figure S1**

**A** Representative immunofluorescence images of spleen samples from HDC-EGFP mice and HDC^-/-^ mice stained with HDC (green) and DAPI (nuclei, blue). Bar, 10 μm. **B** Representative images and quantitative analysis of cardiac function at AMI D7 evaluated by left ventricular ejection fraction (LVEF). WT AMI mice were administrated intraperitoneally daily with pyrilamine (H1Ri, 10 mg/kg) or cimetidine (H2Ri, 10 mg/kg), and HDC^-/-^ AMI mice were administrated with histamine (HA, 4 mg/kg). **C** Representative images of cardiac injury at AMI D7 showed by H&E staining. The infarcted area is marked with black-dotted lines. Bar, 1 mm. **D** Representative immunofluorescence images of megakaryocytes in spleen stained with Ly6G (neutrophil, red), GFP (HDC, green), CD41 (platelets, grey), and DAPI (nuclei, blue). Bar, 10 μm. Graph shows mean ± SEM. P-values were determined by one-way ANOVA with Bonferroni-Dunn correction.


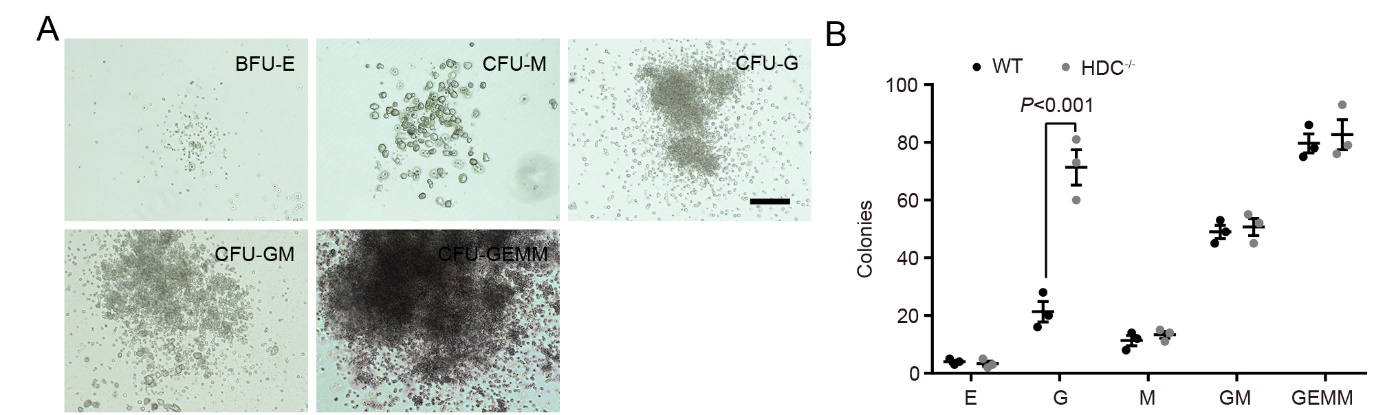


**Figure S2**

**A,B** Colony forming unit (CFU) analysis. **A** Representative images of colonies of erythroid progenitor cells (burst-forming unit-erythroid [BFU-E]), granulocyte and/or macrophage progenitor cells (CFU-granulocyte, macrophage [CFU-GM], CFU-granulocyte [CFU-G] and CFU-macrophage [CFU-M]) and multi-potential progenitor cells (CFU-granulocyte, erythrocyte, macrophage, megakaryocyte [CFU-GEMM]). Bar, 100 μm. **B** Quantitative analysis of colony forming unit. Graph shows mean ± SEM. P-values were determined by one-way ANOVA with Bonferroni-Dunn correction.


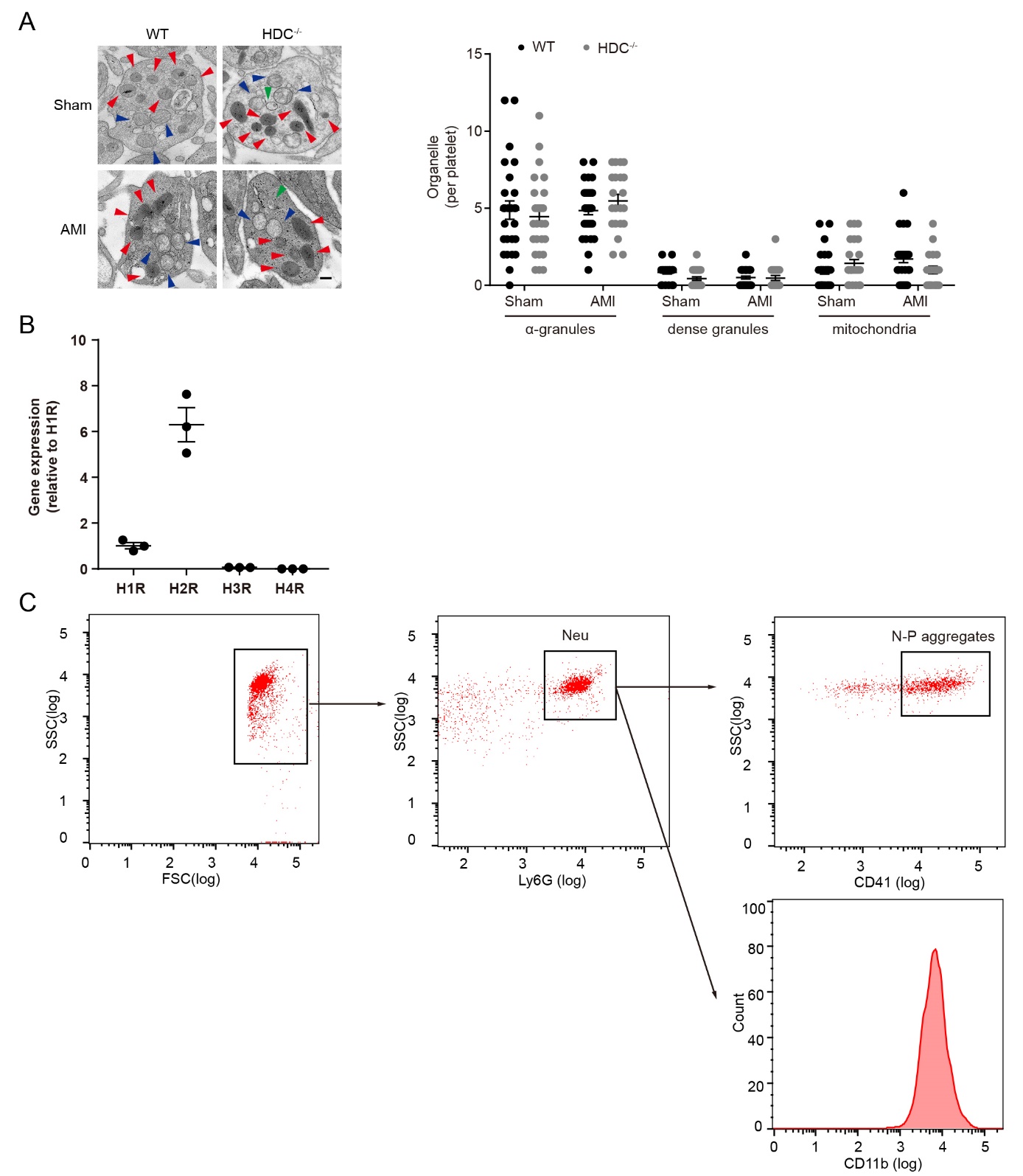


**Figure S3**

**A** Representative ultrastructure images of platelets. Arrowheads show α-granules (red), dense granules (green) and mitochondria (blue). Bar, 200 nm. Graph shows mean ± SEM. P-values were determined by one-way ANOVA with Bonferroni-Dunn correction. **B** Gene expression of histamine receptors in platelets. Expression of actin served as endogenous control. Graph shows mean ± SEM. P-values were determined by one-way ANOVA with Bonferroni-Dunn correction. C Gating strategy of neutrophil-platelet aggregates. Neu, neutrophils. N-P aggregates, neutrophil-platelet aggregates.


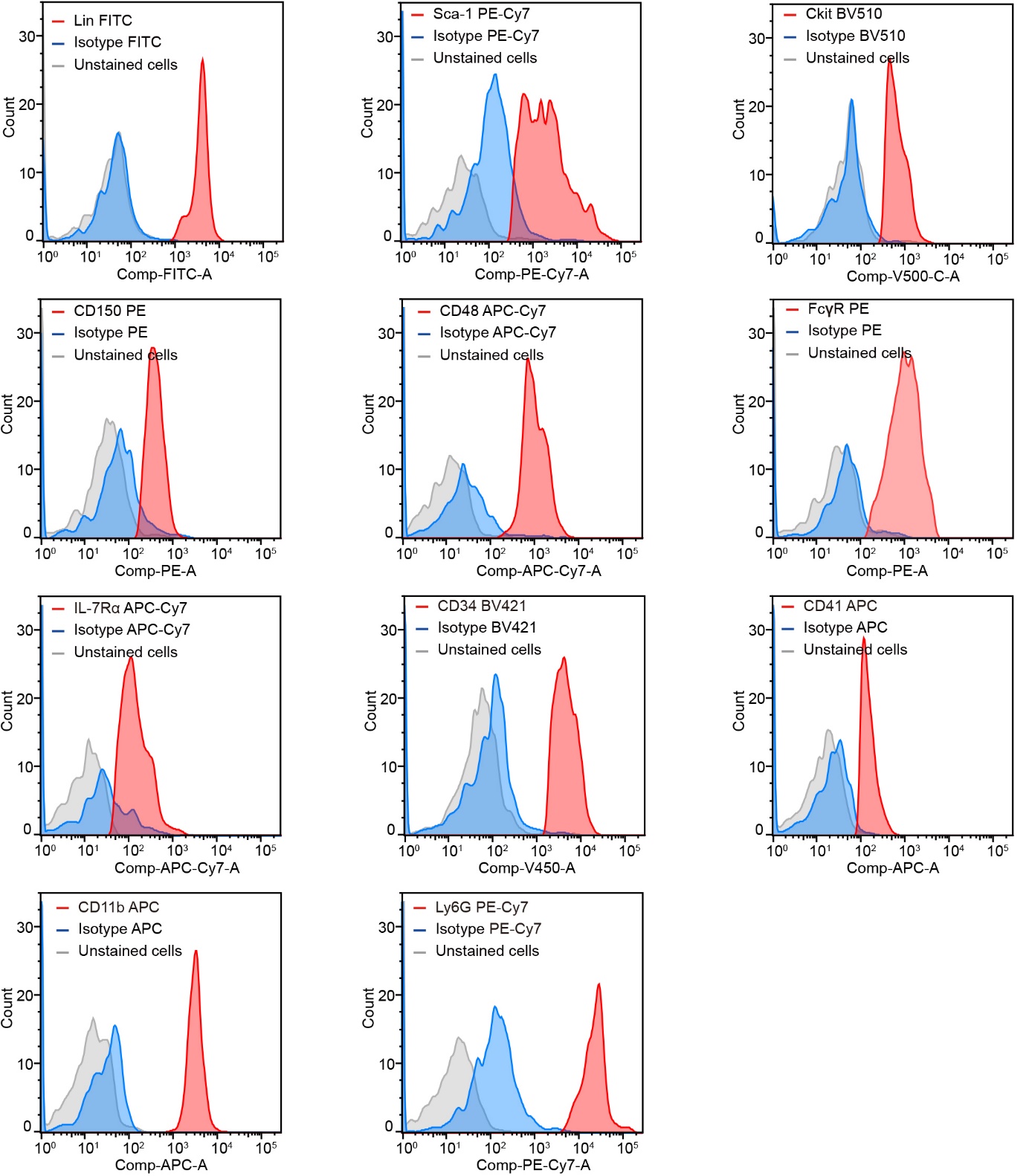


**Figure S4**

Specificity of fluorescence-conjugated antibodies used in flow cytometry of hematopoietic stem and progenitor cells (HPSCs) and neutrophils derived from bone marrow and blood.

**
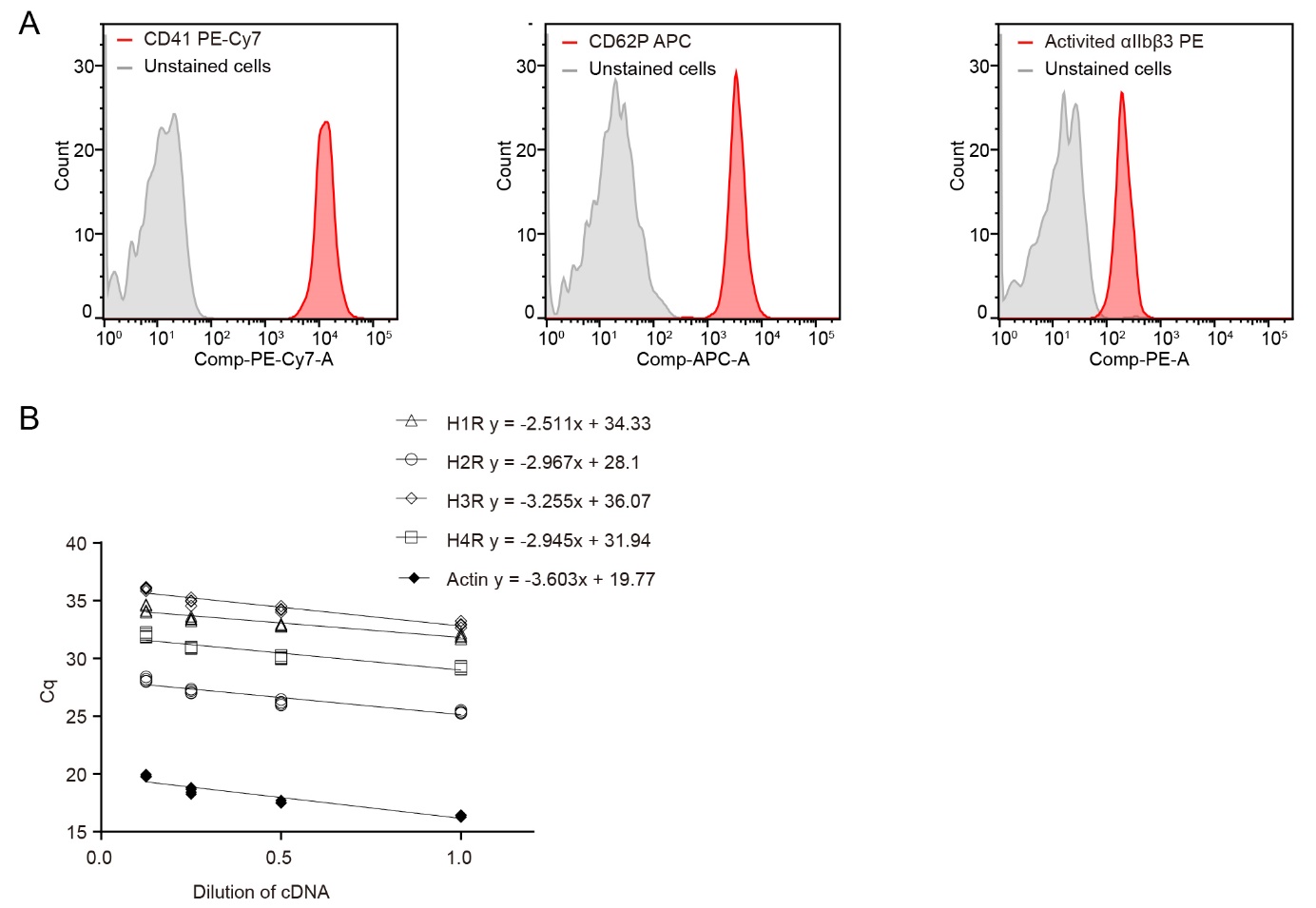
**

**Figure S5**

**A** Specificity of fluorescence-conjugated antibodies used in platelet activation assay. Platelets were stimulated by thrombin (0.1 U/ml). **B** Primer efficiency of H1R, H2R, H3R, H4R and actin. cDNA of bone marrow cells was used as DNA template. The dilution ratios of DNA template were 1/8, 1/4, 1/2 and 1.

**Video legends**

**Video 1** Representative video of intravital microscopy showing platelets (red) and HDC-expressing neutrophils (green) participate in FeCl_3_-induced mesenteric arteriole thrombus. Monitoring of thrombus formation in mesenteric arteriole was initiated 1 min before FeCl_3_ treatment and continuously monitored for 27 min (before treatment 1 min + treatment duration 1 min + thrombus formation 25 min).

**Video 2** Representative video of intravital microscopy in WT→HDC-EGFP mice. Platelets (red), HDC-expressing neutrophils (green).

**Video 3** Representative video of intravital microscopy in HDC^-/-^→HDC-EGFP mice. Platelets (red), HDC-expressing neutrophils (green).
